# Supplementary figures and images for: Oxylipin biosynthetic gene families of Cannabis sativa
Source: PLoS One. 2023 Apr 26;18(4):e0272893. doi: 10.1371/journal.pone.0272893 (PMC10132601; doi:10.1371/journal.pone.0272893)

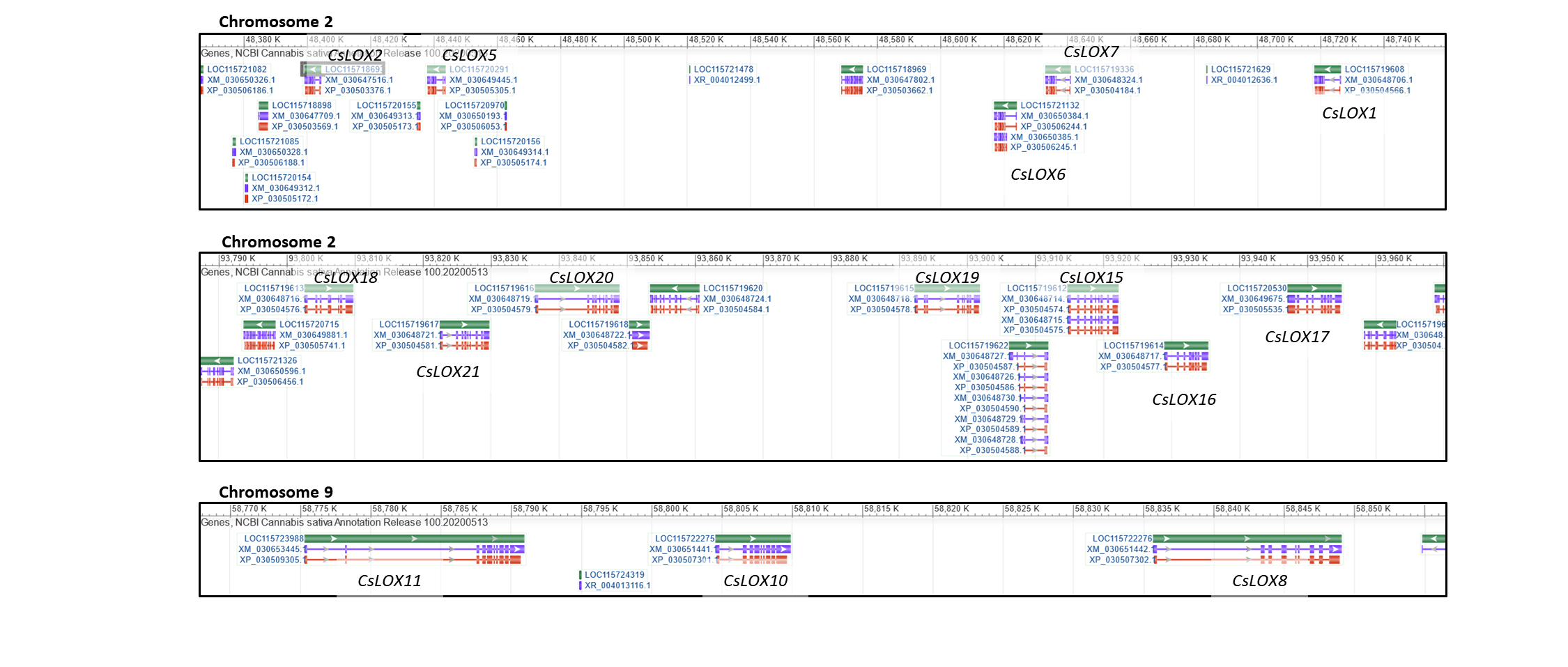

Supplement: S1 Fig — (TIF) [file pone.0272893.s001.tif]

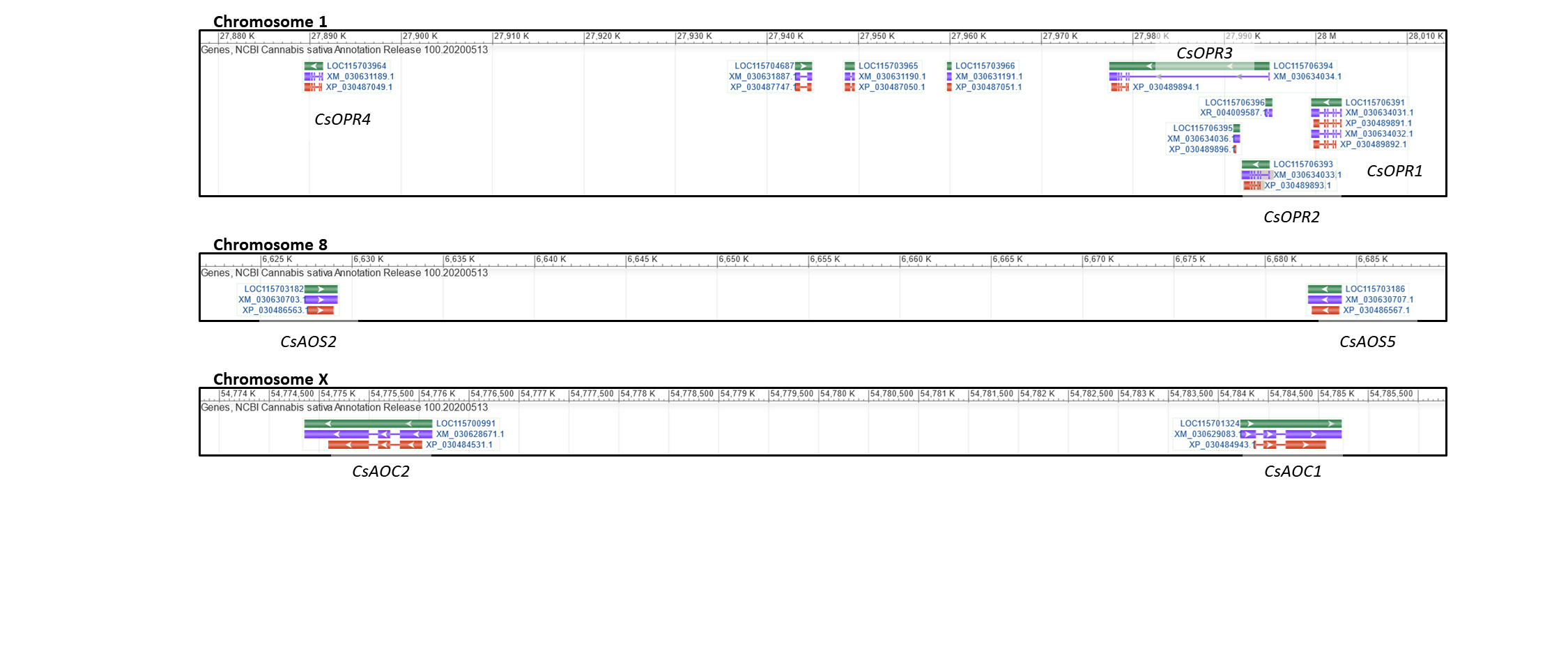

Supplement: S2 Fig — (TIF) [file pone.0272893.s002.tif]

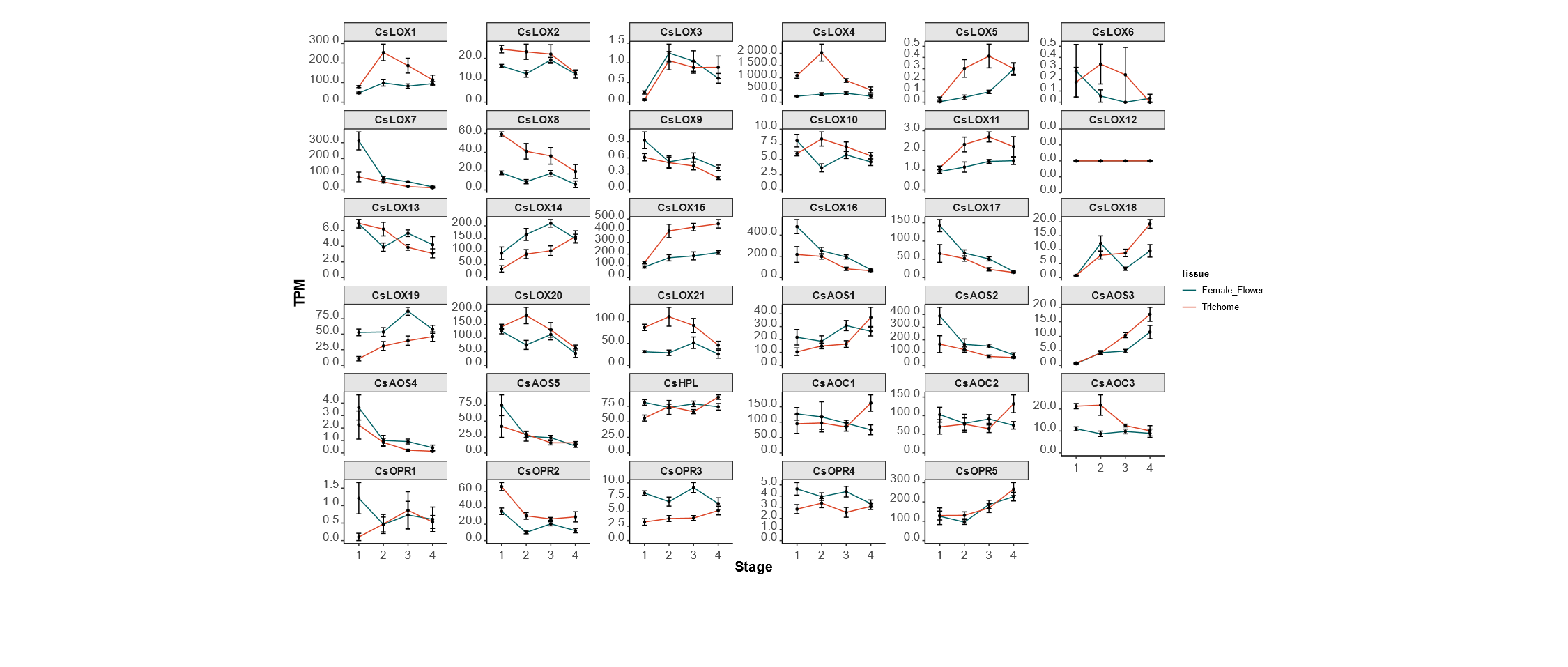

Supplement: S3 Fig — (TIF) [file pone.0272893.s003.tif]
